# Chitosan Upregulates the Genes of the ROS Pathway and Enhances the Antioxidant Potential of Grape (*Vitis vinifera* L. ‘Touriga Franca’ and ’Tinto Cão’) Tissues

**DOI:** 10.3390/antiox8110525

**Published:** 2019-11-03

**Authors:** Rupesh K. Singh, Bruno Soares, Piebiep Goufo, Isaura Castro, Fernanda Cosme, Ana L. Pinto-Sintra, António Inês, Ana A. Oliveira, Virgílio Falco

**Affiliations:** 1 Centro de Química de Vila Real (CQ-VR), Universidade de Trás-os-Montes e Alto Douro (UTAD), Quinta de Prados, 5000-801 Vila Real, Portugal; fcosme@utad.pt (F.C.); aines@utad.pt (A.I.); 2 Departamento de Agronomia, Universidade de Trás-os-Montes e Alto Douro (UTAD), Quinta de Prados, 5000-801 Vila Real, Portugal; bmgoncalves90@gmail.com (B.S.); pgoufo@utad.pt (P.G.); anaolive@utad.pt (A.A.O.); 3 CoLAB Vines&Wines, Associação para o Desenvolvimento da Viticultura Duriense (ADVID), Régia Douro Park, 5000-033 Vila Real, Portugal; 4 Centro de Investigação e Tecnologias Agroambientais e Biológicas (CITAB), Universidade de Trás-os-Montes e Alto Douro (UTAD), Quinta de Prados, 5000-801 Vila Real, Portugal; icastro@utad.pt (I.C.); asintra@utad.pt (A.L.P.-S.)

**Keywords:** chitosan, elicitor, *Vitis vinifera* L., antioxidant activity, secondary metabolites, anthocyanins, ROS pathway, polyphenols, tannins

## Abstract

Chitosan is an environmentally-friendly active molecule that has been explored for numerous agricultural uses. Its use in crop protection is well-known, however, other properties, such as bioactivity, deserve attention. Moreover, the modes of actions of chitosan remain to be elucidated. The present study assessed the levels of total phenolic compounds, the antioxidant potential, and the expression of reactive oxygen species (ROS) scavenging genes in the berries (skins and seeds), leaves, cluster stems, and shoots upon chitosan application on two red grapevine varieties (Touriga Franca and Tinto Cão). The application of chitosan on the whole vine before and after veraison led to the increased levels of polyphenols, anthocyanins, and tannins in Tinto Cão berries, and polyphenols and tannins in Touriga Franca berries, respectively. CUPric Reducing Antioxidant Capacity (CUPRAC) and Ferric Reducing Antioxidant Power (FRAP) assays indicated an increase in the antioxidant potential of berries. With the exception of ascorbate peroxidase (APX), all the ROS pathway genes tested, i.e., iron-superoxide dismutase (Fe-SOD), copper-zinc-superoxide dismutase (Cu/Zn-SOD), catalase (CAT), glutathione reductase (GR), glutaredoxin (Grx), respiratory burst oxidase (Rboh), amine oxidase (AO), peroxidase (POD) and polyphenol oxidase (PPO), were found up-regulated in chitosan-treated berries. Results from the analyses of leaves, stems, and shoots revealed that chitosan not only induced the synthesis of phenolic compounds but also acted as a facilitator for the transfer of polyphenols from the leaves to the berries.

## 1. Introduction

Grapes are non-climacteric berry fruits produced by deciduous and perennial woody vines. For centuries, grapes have had a huge impact on the world’s economy due to their use in winemaking and in a vast variety of food products [1,2]. The quality of grape-derived products depends on the levels of secondary metabolites in the berries, including anthocyanins, tannins, stilbenes, flavonoids, and other phenolic compounds. Although these compounds are known for their antioxidant properties, they are particularly important for the flavor, color, and taste of wines as well as other industrial interests like neutraceuticals and pharmaceutical products [3,4,5]. 

For years now, increasing the levels of secondary metabolites in grape berries has been a major subject of interest worldwide. Several cultural practices have been studied to improve the accumulation of secondary metabolites in grape and wine [1,6,7,8,9,10,11,12]. It was found that vine ‘cordon training’ and pruning led to the increased levels of tannins and anthocyanins in red wine [9]. The total anthocyanin and the total phenolic contents were also higher in Tannat, Malbec, and Xinomavro grapes obtained from vines subjected to cluster thinning, when compared with “not thinned” controls [6,7,8]. Koundouras et al. reported that deficit irrigation caused a substantial increase of the level of anthocyanins in the skins of Cabernet Sauvignon grapes [10]. Subsequent studies in the following years confirmed this finding [11,12]. 

Besides the above-mentioned traditional cultural practices, the use of electrical [1] and chemical [13] elicitors have also been attempted. Elicitor-mediated induction of plant defenses is one of the most effective strategies in enhancing the production of secondary metabolic compounds, and the approach has been tested in various crops [13]. An elicitor is defined as “a compound introduced in small concentration to a living system to promote specific responses” [13]. In plants, several molecules, such as phenylalanine, urea, benzothiadiazole, methyl jasmonate, yeast extract, abscisic acid, and chitosan have been used as elicitors [14,15,16,17,18]. Chitosan, in particular, has received a scrutinized interest giving its efficacy in the crop protection [18,19,20,21,22]. Chitosan is a cheap linear polysaccharide derived from the chitin of fungal cell walls and exoskeleton of arthropods [23,24]. Chitosan is safe, biodegradable, non-allergic, bio-compatible, and the second most abundant renewable carbon source after cellulose [25,26,27,28]. These properties have ensured their wide industrial use [23]. Chitosan was introduced for agricultural purposes in the 1980s. The first report of chitosan as an elicitor was in pea (*Pisum sativum* L.) and tomato (*Solanum lycopersicum* L.) [19]. Its application resulted in an increased accumulation of the phytoalexin pisatin in pea, and anti-nutrient proteinase inhibitors in tomato. Chitosan was later used to induce the accumulation of secondary metabolites in many other plant species. For example, it was demonstrated that foliar application of chitosan enhances tomato fruit weight, productivity, and resistance against fungal pathogens [20,21], improves the yield and vegetative growth of strawberries [29], and the quality and self-life of kiwifruit [30].

There is a worldwide trend to explore the properties of chitosan for sustainable agriculture [25,27,28]. There are, however, only sporadic reports on in-field applications of chitosan on grapevine plants. Spraying of chitosan combined with yeast on vines before harvest reduced the decay of berries during cold storage [31]. In vitro cultured grape plantlets supplemented with a chitosan gel demonstrated improved growth and resistance towards the fungal pathogen *Botrytis cinerea* [22]. Vitalini et al. reported increased levels of aromatic compounds in wines obtained from chitosan-treated vines, as compared to those vines which were treated with conventional fungicides [15]. There was also an improvement in the overall sensory acceptance of wines [15]. Although Portu et al. did not find a substantial impact on the phenolic composition of grapes and wines following chitosan treatment [16]. The above-mentioned studies highlight the necessity of conducting more research on the response of vines concerning the application of chitosan.

Moreover, the mode of action of chitosan is still unknown for several aspects of plant protection and productivity. Chitosan is known to induce plant defense mechanisms by indirectly stimulating the synthesis of secondary metabolites [16,23]. The metabolic pathways regulated by chitosan, which ultimately lead to drought resistance in white clover, have been reported recently [26]. In particular, it was found that chitosan-induced drought resistance is associated with the accumulation of endogenous chitosan, and the enhancement of the ascorbate−glutathione and tricarboxylic acid cycle pathways [26]. In the present study, an additional insight in the mode of action of chitosan is provided by analyzing vines in a tissue-specific manner, after field application of chitosan during and after veraison on *Vitis vinifera* L. ‘Touriga Franca’ and ‘Tinto Cão’. ‘Touriga Franca’ and ‘Tinto Cão’ are the most widely grown varieties of grapevines in the Douro Demarcated Region of Portugal and are very important varieties for Port wine production. Berries’ seeds and skins, stems, leaves, and shoots were analyzed for the total phenolic content (TPC), total anthocyanin content (TAC), total tannin content (TTC), antioxidant potential, and the expression of the reactive oxygen species (ROS) pathway genes.

## 2. Materials and Methods

### 2.1. Plant Material, Treatments, and Sample Collection

*Vitis vinifera* L. ‘Touriga Franca’ and *Vitis vinifera* L. ‘Tinto Cão’ (red varieties), grown in the Quinta de Nossa Senhora de Lourdes vineyard (41°19’ N, 7°44’ W, 500 m above mean sea level), were used in this study. The vineyard is in Vila Real, which is situated in the lower Corgo sub-region of the Douro Demarcated Region of northern Portugal. The vines (420A rootstock) were trained with the Guyot system, spaced 2.3 × 0.9 m, on a grass-covered morainic soil, with 15% of gravel and loamy sand. The trial was set up as a complete randomized block design in three replications (lines), with 12 vines per line (chitosan-treated vines and control vines) in each line. Spraying of crop protectants, weed control, and shoot guiding were the same for all vines.

**Figure 1:**
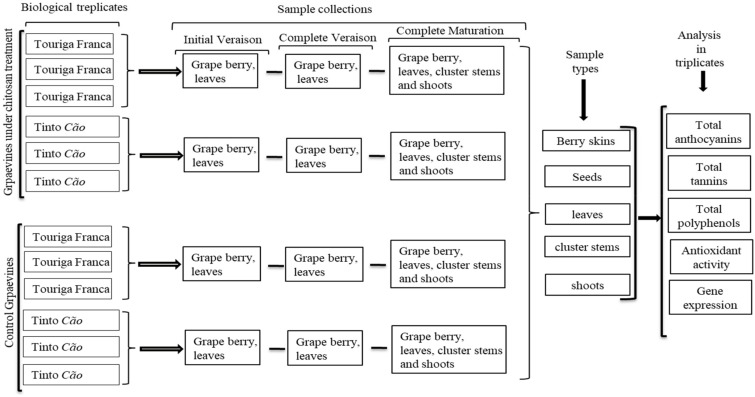
Diagrammatic representation of the complete experimental setup, including sample collection and sample analyses.

Chitosan (molecular weight of 76 kDa and deacetylation degree of 85%) was obtained from Sigma-Aldrich (St. Louis MO) and dissolved in 0.01 M aqueous acetic acid solution to a concentration of 0.1% (w/v). The whole vine, including the fruits, was sprayed with 200 mL of the chitosan solution, using a manual spray lance. Chitosan was applied on the same vines twice, first at the beginning of veraison at the onset of berry ripening, and second at the completion of veraison with all the berries colored red. The meteorological conditions from the beginning of veraison until berry harvesting were as follows: average temperature, humidity, and wind velocity of 23.4 °C, 57.4%, and 6.5 km/h, respectively. Berries and leaves were collected during veraison, after complete veraison, and at complete maturation. Stems and shoots were collected at the complete maturation of the berries, as explained diagrammatically in Figure 1. The same tissues from all 12 vines from a line were pooled to constitute a sample.

### 2.2. Sample Preparation

The leaves were removed from the vines using a scissor. The seeds were manually separated from the berries’ skins. The stems and shoots were chopped in small pieces using a scissor. All processed samples were immediately frozen in liquid nitrogen and stored in –80 °C. Before the analyses, all the samples were freeze-dried and grounded into a fine powder (ca. 0.5 mm size) using a coffee grinder (Q.5321, Qilive, China).

Aqueous ethanol (1.5 mL, 50% *v*/*v*) was added to 100 mg of powdered sample and the contents were mixed in an agitator for 1 h at room temperature (ca. 25 °C). The mixture was then centrifuged at 10,000 rpm for 20 min at 4 °C. The supernatant was transferred to a tube and the residue was re-extracted as described above. Both the supernatants were combined, made up to 3 mL with 50% aqueous ethanol, and stored at −20 °C until further analyses.

### 2.3. Determination of the Total Phenolic Content (TPC) and the Total Anthocyanin Content (TAC)

The TPC and TAC were estimated using a protocol adapted from two previous publications [5,32]. An Aliquot (200 µL) of the supernatant extract obtained in 2.2 was acidified with 3.8 mL of 1.0 M aqueous HCl. The two solutions were mixed by gentle inversion, followed by incubation at 28 °C for 3 h. The absorbances at 520 nm and 280 nm were recorded using a spectrophotometer (Evolution 201, Thermo Scientific). A520 values were converted into malvidin-3-*O*-glucoside contents based on a generic extinction coefficient of malvidin-3-*O*-glucoside [5], and the TAC was expressed as µg of malvidin-3-*O*-glucoside equivalents per mg dry weight. The TPC was calculated and expressed as µg of epicatechin equivalents per mg dry weight, using a calibration curve for epicatechin. 

### 2.4. Determination of the Total Tannin Content (TTC)

The TTC was determined using the methyl cellulose-mediated precipitation method [33,34]. A mixture was prepared by adding 600 µL of 0.04% methyl cellulose to 200 µL of the supernatant extract obtained in Section 2.2. After incubation for 3 min at room temperature, 400 µL of a saturated solution of ammonium sulphate and 0.8 mL of deionized water were added and the contents mixed by inversion. A control mixture was also prepared, but with deionized water (600 µL) instead of 0.04% methyl cellulose. After incubation for 10 min at room temperature, the mixtures were centrifuged for 5 min at 4,000 rpm (4 °C). The resulting supernatant was read at 280 nm. The TTC was obtained by subtracting the A280 nm extract and the A280 nm control. The tannin value was converted into epicatechin equivalents (µg per mg dry weight) with the aid of a calibration curve.

### 2.5. Determination of the Antioxidant Activity Using the DPPH, CUPRAC, and FRAP Assays

#### 2.5.1. Sample Preparation

Powdered lyophilized samples (40 mg) were extracted with 1 mL of 70% methanol. After incubation at 70 °C for 30 min, the solution was cooled at room temperature and centrifuged at 11,000 rpm for 20 min in order to obtain the supernatant used for the analyses.

#### 2.5.2. DPPH (2,2-diphenyl-1-picrylhydrazyl) Assay

The DPPH assay used in this study was a modification of previous methods [35,36,37]. A 4% DPPH solution was prepared in 95% ethanol. The supernatant obtained in 2.5.1 (15 µL) was added to the DPPH solution in a 96-wells microplate to a final volume of 300 µL. The decrease in absorbance at 517 nm (*ABS sample*) was measured with a microplate reader, against a blank prepared with 70% methanol instead of the supernatant (*ABS blank*). The DPPH antioxidant activity was expressed as the percentage of the remaining DPPH in the reaction medium (% AA) using Equation (1). 

(1) $$\%AA=\left(ABS\;blank-\left(\frac{ABS\;sample}{ABS\;blank}\right)\right)\times 100$$

#### 2.5.3. FRAP (Ferric Reducing Antioxidant Power) Assay

The FRAP assay was modified from [38,39]. A portion of an aqueous 10 mM solution of 2,4,6-Tri(2-pyridyl)-s-triazine in 40 mM HCl was mixed with the same volume of 20 mM FeCl_3_.6H_2_O and 10 times higher volume of acetate buffer of pH 3.6 (3.1 g sodium acetate and 16 mL acetic acid per L). The mixture was incubated at 37 °C for 20 min. A portion (900 µL) of the ferric tripyridyltriazine (Fe^3+^-TPTZ) mixture and 20 µL of supernatant were diluted up to 1000 µL with deionized water and incubated for 10 min, and the absorbance was measured at 593 nm. A standard curve was prepared using fresh FeSO_4_ solutions, and the ferric reducing power was expressed as mg FeSO_4_ equivalents per mg dry weight. 

#### 2.5.4. CUPRAC (CUPric Reducing Antioxidant Capacity) Assay

The CUPRAC assay was carried out as described by [40]. Briefly, 0.2 mL of supernatant was added with 0.5 mL of 2 × 10^−3^ M Cu(II) chloride, 0.5 mL ethanol, 1 mL of 7.5 × 10^−3^ M neocuproine (Nc), 1 mL of 1 M ammonium acetate (NH_4_/Ac) buffer pH 7.0, and 0.8 mL ethanol. After 30 min of incubation at room temperature, the absorbance at 450 nm was recorded against a water blank. The standard calibration curve of trolox was constructed as absorbance vs. concentration, and the antioxidant activity expressed as mg trolox equivalents per mg dry weight. 

### 2.6. RNA Extraction, cDNA Synthesis, and qRT-PCR Analysis

Total RNA was extracted from frozen tissues harvested after complete maturation of the vines, using the CTAB (cetyl trimethyl ammonium bromide) buffer as described in [41]. Total RNA was extracted by using the RNeasy Mini Kit (Qiagen, Germany) and a DNase treatment was performed respectively. The integrity of the total RNA was verified by running samples on 1.0% denaturing agarose gels, followed by quantification with a ND-1000 Spectrophotometer (NanoDrop Technologies, Wilmington, DE) at wavelengths of 230 and 280 nm. A total of 1 µg of DNase-treated RNA was used for cDNA synthesis, using the First Strand cDNA Synthesis Kit (Solis Biodyne, Tartu, Estonia). Specific internal primers (Table 1) were designed using Primer3 online tool (http://primer3.ut.ee/). Quantitative real-time PCR (qRT-PCR) was performed to estimate the relative expression of 10 selected genes of the ROS pathway. Each reaction contained 5 × HOT FIREPol^®^ EvaGreen^®^ qPCR Mix Plus (Solis Biodyne, Tartu, Estonia), 200 nM of each primer, 1 µL 10 × diluted cDNA sample, and nuclease-free water to a final volume of 20 µl. Reactions were carried out on PicoReal-Time Thermal Cycler (PRO 96-1500-512, Thermo Scientific, Ratastie, Finland). The PCR program was: 95 °C for 12 min (initial denaturation), followed by 40 cycles of heating at 95 °C for 15 s (denaturation), 52 °C for 20 s (annealing), and 72 °C for 20 s (extension). No final extension was carried out. The software PicoReal 2.0 was used to analyze the data. The relative mRNA level for each gene was calculated as RQ values (relative quantification by using PicoReal 2.0 tool), using actin as the internal reference gene.

### 2.7. Statistical Analyses

Statistical analyses were carried out using JMP 11.0 (SAS Institute Inc, Cary, NC). For each vine tissue, the effects of chitosan application on the TPC, TAC, and TTC during veraison, after veraison and at maturity were assessed using a two-way analysis of variance (ANOVA). The comparison among means was determined according to Turkey’s test. When only two means were available, an independent *t*-test was used. Antioxidant activities (DPPH, FRAP, CUPRAC) and gene expression data for each tissue were subjected to an independent *t*-test analysis. All analyses were performed in triplicates (technical replicates) and results are expressed as mean ± standard deviation of data recorded from three independent samples (biological replicates). Differences between means were considered meaningful at *p* < 0.05 and represented by different letters for ANOVA and asterisks for the *t*-test.

## 3. Results

### 3.1. Effect of Chitosan Application on Polyphenols in Grapevine

Grape seeds, skins, and juice contain a substantial amount of polyphenolic compounds which explain the health benefits of grape-based nutraceutical products [23]. In this study, polyphenols accumulated more in the seeds than in the skins of ‘Tinto Cão‘, and more in the skins than in the seeds of ‘Touriga Franca‘ (Table 2). At the first and second harvesting times, the TPC increased in leaves, skins, and seeds collected from vines treated with chitosan, in comparison to samples from control vines: increases of 24.18%, 29.51%, and 8.96% in ‘Touriga Franca‘ during veraison; 31.80%, 22.83%, and 13.31% in ‘Touriga Franca‘ after veraison; 10.53%, 23.01%, and 3.40% in ‘Tinto Cão‘ during veraison; 2.09%, 20.40%, and 18.17% in ‘Tinto Cão‘ after veraison (Table 2). At maturity, the TPC was also higher in the skins of chitosan-treated vines than in those of control vines, with 8.96% and 25.71% increases in ‘Touriga Franca‘ and ‘Tinto Cão‘, respectively. An increase was also recorded in the leaves of ‘Touriga Franca‘ (41.44%), with no change in the leaves of ‘Tinto Cão‘. At complete maturation, the TFC was higher in the stems from chitosan-treated vines than those of control vines, with increases of 28.73% and 31.75% for ‘Touriga Franca‘ and ‘Tinto Cão‘, respectively. The application of chitosan had no effect in the TPC in the seeds and shoots of the two varieties (Table 2).

### 3.2. Effect of Chitosan Application on Anthocyanins in Grapevine

Anthocyanins are a special class of polyphenols involved in resistance against diseases in the leaves and shoots, and responsible for the color of berries and wines [1]. Table 3 shows that anthocyanins accumulated mainly in the skins of the fruits, as compared to the seeds where they were usually not detected. After application of chitosan, the TAC increased in the skins of ‘Touriga Franca‘ and ‘Tinto Cão‘ during veraison by 35.27% and 56.02%, respectively and after veraison by 17.42% and 10.51%, respectively. At complete maturation, a 22.33% increase in TAC was also recorded in the skins of ‘Tinto Cão‘, in comparison with the skins from control vines. On the contrary, the TAC decreased by 10.55% in the skins of ‘Touriga Franca‘ at maturity (Table 3). Before maturation, the TAC also increased in the leaves of chitosan-treated vines, 28.21% in ‘Touriga Franca‘ and 22.37% in ‘Tinto Cão‘ during veraison, and 37.50% and 14.93% after veraison. At complete maturation, the TAC was also higher in ‘Touriga Franca‘ leaves (35.90% increases) treated with chitosan, while no change was recorded for ‘Tinto Cão‘ (Table 3). At complete maturation, more anthocyanins accumulated in the stems of chitosan-treated vines than in those of control vines, with 113.76% and 16.04% increases of TAC in ‘Touriga Franca‘ and ‘Tinto Cão‘, respectively. The TAC of the shoots was not affected by the application of chitosan (Table 3).

### 3.3. Effect of Chitosan Application on Tannins in Grapevine

Tannins contribute to the astringency of grapes and, in combination with anthocyanins, define the stability of wine color [34]. In this study, tannins tended to accumulate more in the seeds than the skins, although this depended on the growth stage of the vines (Table 4). At all harvesting times, the TTC increased in the skins and seeds collected from vines treated with chitosan, as compared to control vines: 14.18% and 325.15% in ‘Touriga Franca‘, 9.61% and 9.23% in ‘Tinto Cão‘ during veraison; 37.48% and 159.02% in ‘Touriga Franca‘, 52.62% and 21.32% in ‘Tinto Cão‘ after veraison; 20.46% and 32.12% in ‘Touriga Franca‘, 48.46% and 6.01% in ‘Tinto Cão‘ at complete maturation. The effect of chitosan on the TTC in the leaves was varied among varieties. A decrease in the leaves of ‘Touriga Franca‘ during (11.62%) and after (9.73%) veraison and an increase at complete maturation (90.33%); and the exact opposite for ‘Tinto Cão‘ i.e., an increase during (36.14%) and after (23.71%) veraison, and a decrease after complete maturation (11.98%) (Table 4), was observed. A variety effect was also observed at maturity for the stems, with chitosan application leading to a lower TTC in ‘Touriga Franca‘ (35.12 decreases) and a higher TTC in ‘Tinto Cão‘ (37.01% increase), in comparison of the stems of control vines. Treatment of vines with chitosan led to an increased TTC in the shoots of both varieties, with 114.48% and 23.48% increases in ‘Touriga Franca‘ and ‘Tinto Cão‘, respectively (Table 4).

### 3.4. Effect of Chitosan Application on the Antioxidant Potential of Grapevine

**Figure 2:**
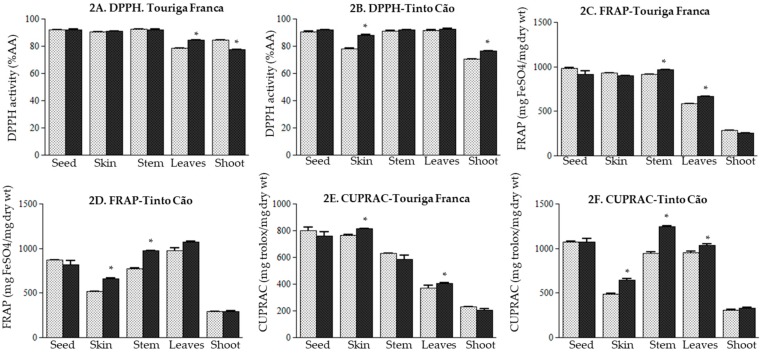
(**A**,**B**) 2,2-diphenyl-1-picrylhydrazyl activity (DPPH), (**C**,**D**) Ferric Reducing Antioxidant Power (FRAP), (**E**,**F**), Copper Reducing Antioxidant Capacity (CUPRAC) in the tissues of grapevine after application of chitosan before and after veraison. Tissues were collected at complete maturation of the berries. Gray column = control samples; black column = chitosan-treated samples. All data were obtained from three biological replicates; standard deviations and the levels of significance (*p* < 0.05; *t*-test) are represented by error bars and asterisks above the columns, respectively.

In this study, three assays were used for assessing the antioxidant potential of grapevine samples, namely DPPH, FRAP, and CUPRAC [35,38,40]. Data in Figure 2 shows that chitosan application did not affect the antioxidant potential of the seeds of the two varieties studied. Among skins, the antioxidant potential remained unchanged for ‘Touriga Franca‘ (Figure 2A,C,E), while it increased for ‘Tinto Cão‘: 11.54%, 27.06%, and 32.51% for the DPPH (Figure 2B), FRAP (Figure 2D) and CUPRAC (Figure 2F), respectively. With respect to the control, FRAP and CUPRAC increased in the stems of ‘Touriga Franca‘ (5.30% and 6.10%, respectively; Figure 2C,E) and ‘Tinto Cão‘ (25.74% and 31.53%, respectively; Figure 2D,F) following chitosan application, while no change was recorded for the DPPH (Figure 2A,B). Chitosan-treated leaves exhibited more antioxidant activities than control leaves: for ‘Touriga Franca‘, 7.72%, 13.60%, 10.32% increases for the DPPH (Figure 2A), FRAP (Figure 2C) and CUPRAC (Figure 2E), respectively, were recorded. For ‘Tinto Cão‘, 1.23% (*p* ˃ 0.05), 9.70%, 8.70% increases for the DPPH (Figure 2B), FRAP (Figure 2D) and CUPRAC (Figure 2F), respectively, were recorded; however, these increases were non-significant. Chitosan application had no effect on the FRAP and the CUPRAC of the shoots of both varieties (Figure 2C–F). In terms of DPPH, a decreased activity (8.33%) was recorded in the shoots of chitosan-treated ‘Touriga Franca‘ (Figure 2A), while an increased activity (11.80%) was recorded in the shoots of chitosan-treated ‘Tinto Cão‘ (Figure 2B).

### 3.5. Effect of Chitosan Application of the Expression of Genes of the ROS Producing and Scavenging Pathway

A qRT-PCR was used for assessing the effect of chitosan on 10 genes of the ROS pathway in grapevine tissues collected at complete berry maturation. 

**Figure 3:**
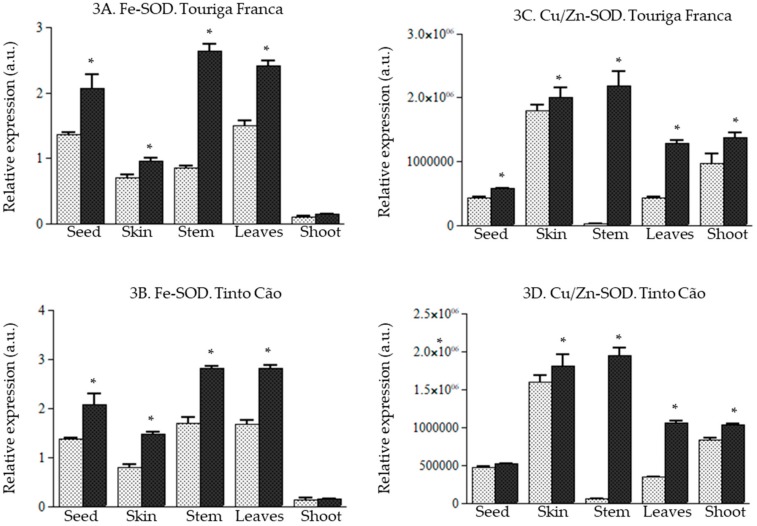
Effect of chitosan on *Fe-SOD* (Fe superoxide dismutase) and *Cu/Zn-SOD* (Cu/Zn superoxide dismutase) genes in the tissues of grapevine (**A**). relative expression of *Fe-SOD* gene in Touriga Franca, (**B**). relative expression of *Fe-SOD* gene in Tinto Cão, (**C**). relative expression of *Cu/Zn-SOD* gene in Touriga Franca, (**D**). relative expression of *Cu/Zn-SOD* gene in Tinto Cão. Tissues were collected at complete maturation of the berries. Gray column = control samples; black column = chitosan-treated samples. All data were obtained from three biological replicates; standard deviations and the levels of significance (*p* < 0.05; *t*-test) are represented by error bars and asterisks above the columns, respectively.

With one exception (*Fe-SOD* remained unaffected in the shoots of ‘Tinto Cão‘), chitosan upregulated or tended to upregulate the expression of the genes *Cu/Zn-SOD* and *Fe-SOD* in all tissues of ‘Touriga Franca‘ and ‘Tinto Cão‘ (Figure 3). The most important changes were observed for *Fe-SOD* in the stems of ‘Touriga Franca‘ (2.0-fold increase; Figure 3A); and for *Cu/Zn-SOD* in the leaves of ‘Touriga Franca‘ (3.3-fold increase), leaves of ‘Tinto Cão‘ (3.0-fold increase), stems of ‘Touriga Franca‘ (7.4-fold increase), and stems of ‘Tinto Cão‘ (7.1-fold increase) (Figure 3C,D). 

**Figure 4:**
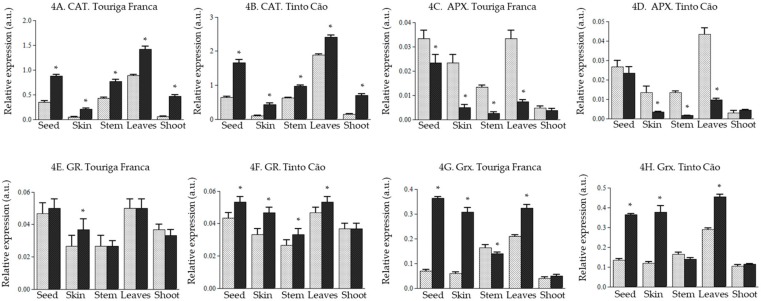
Effect of chitosan on *CAT* (catalase), *APX* (ascorbate peroxidase), *GR* (gluthione reductase), and *Grx* (glutaredoxin) genes in the tissues of grapevine (**A**). relative expression of *CAT* gene in Touriga Franca, (**B**). relative expression of *CAT* gene in Tinto Cão, (**C**). relative expression of *APX* gene in Touriga Franca, (**D**). relative expression of *APX* gene in Tinto Cão, (**E**). relative expression of *GR* gene in Touriga Franca, (**F**). relative expression of *GR* gene in Tinto Cão, (**G**). relative expression of *Grx* gene in Touriga Franca, (**H**). relative expression of *Grx* gene in Tinto Cão. Tissues were collected at complete maturation of the berries. Gray column = control samples; black column = chitosan-treated samples. All data were obtained from three biological replicates; standard deviations and the levels of significance (*p* < 0.05; *t*-test) are represented by error bars and asterisks above the columns, respectively.

CAT expression was also upregulated in all the tissues of grapevine after application of chitosan, with 1.3 to 7.6-fold increases recorded. The magnitude of the change was particularly important in the skins (3.2 and 4.3-fold for ‘Touriga Franca‘ and ‘Tinto Cão‘, respectively) and the shoots (7.6 and 5.0-fold for ‘Touriga Franca‘ and ‘Tinto Cão‘, respectively) (Figure 4A,B). A varietal effect was observed for *GR* expression, which following chitosan application, was upregulated in the tissues of ‘Tinto Cão‘ (with the exception of a non-significant effect in the shoots), and remained unchanged in the tissues of ‘Touriga Franca‘ (with the exception of a 1.3-fold increase in the skins) (Figure 4E,F). For both varieties, Grx expression was upregulated in the seeds, skins, and leaves, downregulated in the stems, and remained unaffected in the shoots. The highest changes were recorded in the berries, with 5.1-fold and 5.0-fold increases in the skins and seeds of ‘Touriga Franca‘ (Figure 4G), and 3.0-fold and 2.7-fold increases in the skins and seeds of ‘Tinto Cão‘ (Figure 4H). In contrast to *CAT*, *GR*, and *Grx*, the expression of *APX* was downregulated in all the tissues of both varieties upon chitosan treatment, although a non-significant effect was observed for the shoots (Figure 4C,D). The highest decreases were recorded in the skins and leaves (4.0–4.5-fold changes) and in the stems (5.2–8.1-fold changes). 

**Figure 5:**
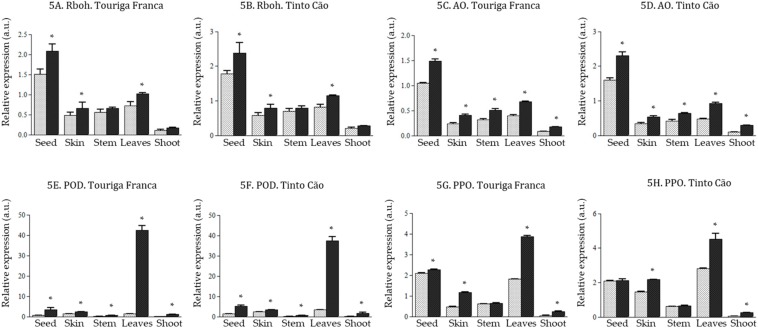
Effect of chitosan on *Rboh* (respiratory burst oxidase), *AO* (amine oxidase), *POD* (peroxidase), and *PPO* (polyphenol oxidase) genes in the tissues of grapevine (**A**). relative expression of Rboh gene in Touriga Franca, (**B**). relative expression of Rboh gene in Tinto Cão, (**C**). relative expression of *AO* gene in Touriga Franca, (**D**). relative expression of *AO* gene in Tinto Cão, (**E**). relative expression of *POD* gene in Touriga Franca, (**F**). relative expression of *POD* gene in Tinto Cão, (**G**). relative expression of *PPO* gene in Touriga Franca, (**H**). relative expression of *PPO* gene in Tinto Cão). Tissues were collected at complete maturation of the berries. Gray column = control samples; black column = chitosan-treated samples. All data were obtained from three biological replicates; standard deviations and the levels of significance (*p* < 0.05; *t*-test) are represented by error bars and asterisks above the columns, respectively.

Following chitosan application, there was an upregulation of the expression of *AO* (1.4-3.0-fold increases) and *POD* (1.4–31.5-fold increases) genes in all tissues of ‘Touriga Franca‘ and ‘Tinto Cão‘ (Figure 5C–F). In particular, the expression of *POD* was 31.5 and 11.0 times higher in the leaves of chitosan-treated ‘Touriga Franca‘ and ‘Tinto Cão‘, respectively, compared to that in the leaves of control vines (Figure 5E,F). The genes *Rboh* (Figure 5A,B) and *PPO* (Figure 5G,H) were also studied upon chitosan application and, with few exceptions of non-significance (*Rboh* in the stems and shoots of both varieties and POP in the stems and seeds of ‘Tinto Cão‘), all showed an upregulation in the tissues of ‘Touriga Franca‘ and ‘Tinto Cão‘ i.e., 1.3–1.4-fold increase for *Rboh* and 1.5–5.0-fold increase for *PPO*, with the highest changes noticed in the shoots. 

## 4. Discussion

Improved fruit yield and quality is a high priority in vitiviniculture, and the use of elicitors holds the potential to help achieve this aim, while decreasing the reliance on pesticides and fertilizers. Previous experiments have instructively illustrated the feasibility of using elicitors to raise the levels of secondary metabolites in grapes. In the previous studies by Portu et al. solutions of methyl jasmonate (10 mM) and chitosan (0.03% in acetic acid 0.01 M) were sprayed over the leaves of *Vitis vinifera* L. ´Tempranillo´ (200–400 mL per vine) at veraison, and a week later, methyl jamsoate was applied which led to an increased accumulation of anthocyanins and stilbenes in the grape and wine, while chitosan treatment did not have a substantial impact on phenolic compounds [16,42]. There is, however, a report that the synthesis of anthocyanins and stilbenes is promoted by chitosan (50 µg/mL in 0.1% v/v acetic acid) in *Vitis vinifera* L.´Barbera´ grape cell suspensions [43]. For *Vitis vinifera* L.´Cabernet Sauvignon´, application of chitosan in three weekly intervals from fruit set to harvest had no effect on the TPC of the grapes, and caused a reduction in extraction of phenolics into the wine during vinification [44]. Thus, chitosan efficacy needs to be validated for each variety/clone in consideration with the dosage, timing, and mode of application. In this study, these variables were studied with a view to establishing the most appropriate conditions for ensuring high levels of phenolic compounds in all tissues. Thus, the whole vine was sprayed with a solution of 0.1% (w/v) chitosan in 0.01 M aqueous acetic acid before and after veraison. 

The data in Table 2 and Table 3 show that during veraison, after veraison, and at complete maturation, chitosan raised the TPC and the TAC of the leaves and berry’s skins of ´Touriga Franca´ and ´Tinto Cão´, with one exception of slight decrease in TAC in the skins of ´Touriga Franca´ at the maturity. The TTC increased in the seeds of chitosan-treated ´Touriga Franca´ and ´Tinto Cão´ at all harvesting times, while an opposite response was observed for the leaves (Table 4), suggesting a varietal dependency. Chitosan is known to stimulate the activity of phenylalanine ammonia lyase, suggesting an induction of the phenylpropanoid pathway [45,46], which would explain the TPC and TAC in this study. The varietal difference observed for the TTC in the leaves might be explained by a preferential activation of the enzymes responsible for anthocyanin synthesis when chitosan is applied on ´Touriga Franca´ leaves, limiting the substrates for the production of tannins [47]. The positive effects of chitosan on the TPC, TAC, and TTC indicate that the treatment could also be regarded as a useful methodology in increasing resistance to some pathogens, as observed with grey mould [45] and powdery mildew infections [18].

Despite increased TPC, TAC and TTC in the berry’s skins at maturity following chitosan application, the antioxidant potential measured by DPPH, CUPRAC and FRAP increased in ´Tinto Cão´, and remained unchanged in ´Touriga Franca´ (Figure 2), which again indicates a varietal dependency. The synthesis and accumulation of phenolic compounds in plants in response to the environmental conditions and cultural practices, are dependent on various factors including the cultivars [2,4,48,49]. This may contribute towards an increased or decreased antioxidant potential [50]. Indeed, chitosan and its formulations are reported to improve and/or reduce the antioxidant potential depending on the crop studied e.g., maize, soybean, jaborandi, Thymus and tobacco [51,52,53,54]. Overall, there was only a loose correlation between TPC/TAC/TTC and the DPPH. On the other hand, a good correlation was found between TPC/TAC/TTC and FRAP/CUPRAC in the stems and the leaves (data not shown).

ROS, such as O_2_·^−^ (superoxide), are free radicals derived from oxygen that is continuously produced during normal aerobic respiration in plants or in response to injury and can be unfavorable to plant fitness [55,56,57,58,59]. Several endogenous antioxidants are produced by plants to slow or stop the production of ROS. SOD (including *Cu/Zn-SOD* and *Fe-SOD*) plays a major role in scavenging ROS in several plants [60,61,62,63], including grapevine [64,65]. The present study demonstrated an upregulation of the SOD gene upon chitosan application. Overall, there was an upregulation of genes of the ROS pathway (*Fe-SOD, Cu/Zn-SOD, CAT, GR, Rboh, AO, PPD,* and *PPO*) in all the tissues of both varieties, with the exception of *APX* in all tissues and *Grx* in the stems of both varieties (Figure 3, Figure 4 and Figure 5). Notably, *POD* increased following application of chitosan, 51.5 and 11.0-fold in the leaves of ´Touriga Franca´ and ´Tinto Cão´, respectively (Figure 5E), suggesting a crucial role for *POD* in the ROS pathway. Recently, it was reported that maize plants are able to cope with drought, following application of chitosan because of the potentiation of the activities of the antioxidant enzymes SOD, *CAT*, *APX*, *GR* and guaiacol peroxidase [46]. This shows that chitosan may induce defense responses in grapevine by activating the ROS pathway, as suggested in some earlier studies [66,67,68,69]. Exogenous chitosan reportedly interacts with chitin synthase, and chitin deacetylase in plants, to produce chitosan oligomers that are responsible for signal perception in cells, leading to the activation of various genes, including genes of the ROS pathway [50,70,71]. Elicitor molecules have been used to stimulate a broad range of responses including defense mechanism [72] in field conditions as well as in vitro cultures for elicitation of secondary metabolites [73,74,75,76]. However, it was reported earlier that acetic acid alone itself may exert some effect [77]. This aspect will be included in the next study as an extra control sample.

A tissue-specific analysis of the response of grapevine to chitosan in this study shows that chitosan might also act by influencing the transport of secondary metabolites in the vine. At maturity, the TPC, and the TAC remained unchanged in the shoots (Table 2 and Table 3), suggesting that shoots are not implicated in the transport of phenolic acids and anthocyanins to the berries. Instead, chitosan seems to stimulate the transfer of leaf anthocyanins to the berries via the stems. At maturity, that transfer seems complete in the case of ´Tinto Cão´, since the TAC and TPC increased in the stems and skins, but remained unchanged in the leaves; and incomplete in the case of ´Touriga Franca´ since a high TAC and TPC is still measured in the leaves. The response of the TTC in the leaves depended on the variety and the harvesting time (Table 4), suggesting that leaf tannins are not transported to the berries. Instead, shoot tannins seem to be transferred to the berries. Finally, the data in Table 2, Table 3 and Table 4 and Figure 2 shows that there is a prospect for adding values to grapevine stems and leaves via the application of chitosan. In general, the levels of phenolics and the antioxidant potential were constantly high in the stems and leaves of both varieties at all harvesting times.

## 5. Conclusions

Present study steps ahead towards a better understanding of chitosan–*Vitis vinifera* L. interaction and elicitation mechanism at the molecular level and suggests its application for value addition in viticulture practices.

## Figures and Tables

**Table 1:** Primer sequences for the genes of the reactive oxygen species pathway analyzed in this study.

Gene		Primer Sequence
*Fe-SOD*	Superoxide dismutase	F 5′ CCTTTGTGAACCTAGGCGAACC 3′ R 5′ TGGCCGGGTTAGCTTGAACTC 3′
*Cu/Zn-SOD*	Superoxide dismutase	F 5′ AGATTGGCATGTGGTGTTGTTG 3′ R 5′ ACTCCCACATTACCCAACAACA 3′
*CAT*	Catalase	F 5′ GGTGTTCACACCTTCACTCT 3′ R 5′ GAGATCCTGAGTAGCATGACTG 3′
*APX*	Ascorbate peroxidase	F 5′ ATCTGGTGGTCATACTCTGG 3′ R 5′ TCTAGGAGAGCCTTGTCTGA 3′
*GR*	Glutathione reductase	F 5′ AATACTAGGGGGAGGGTACA 3′ R 5′ GTTGTCCTTGGATGCAGA 3′
*Grx*	Glutaredoxin	F 5′ CTGCTCTCACAGCTAAAAGC 3′ R 5′ CATCACAGAGTCACATCCAC 3′
*Rboh*	Respiratory burst oxidase	F 5′ CTGATTCTCAGTAGGAACTGGT 3′ R 5′ GAGGTTTGGTCATGTATAGTGC 3′
*AO*	Amine oxidase	F 5′ GTGAATCCGAACAAGAGAAC 3′ R 5′ AGACTTATTGTAGGGTGTGACC 3′
*POD*	Peroxidase	F 5′ CCTGATCCAACACTAGATGC 3′ R 5′ GTCTGACTGAAGAAGTCCAGAG 3′
*PPO*	Polyphenol oxidase	F 5′ CTAGAACTCCAGGTTCATGC 3′ R 5′ GCTTCTCGTCGTAGAGTGAT 3′

**Table 2:** The total phenolic content.

Variety/Tissue	Harvesting Time/Treatment					
	During Veraison		After Veraison		At Complete Maturation	
	Control	Chitosan	Control	Chitosan	Control	Chitosan
‘Touriga Franca’						
berry seeds	109.69 ± 5.04 b	119.27 ± 6.58 a	89.95 ± 5.13 c	101.92 ± 2.47 b	94.67 ± 2.26 c	93.65 ± 5.10 c
berry skins	105.54 ± 2.73 c	136.69 ± 5.40 a	110.61 ± 4.67 c	135.86 ± 5.37 a	112.70 ± 4.23 c	122.80 ± 1.92 ab
cluster stems	NA	NA	NA	NA	88.90 ± 5.55 b	114.44 ± 5.78 a
leaves	70.28 ± 6.27 c	87.27 ± 1.70 a	63.11 ± 0.56 d	83.18 ± 0.86 a	55.65 ± 1.09 e	78.71 ± 1.48 b
shoots	NA	NA	NA	NA	37.23 ± 2.36 a	34.09 ± 0.64 a
‘Tinto Cão‘						
berry seeds	192.64 ± 5.34 b	199.19 ± 2.64 a	154.15 ± 1.86 d	182.14 ± 5.54 c	146.32 ± 4.25 e	144.05 ± 1.23 e
berry skins	80.50 ± 3.61 f	99.03 ± 2.14 e	112.24 ± 5.07 d	135.13 ± 0.60 b	129.95 ± 4.17 c	163.36 ± 9.57 a
cluster stems	NA	NA	NA	NA	158.61 ± 5.28 b	208.97 ± 8.37 a
leaves	223.28 ± 10.10 b	246.79 ± 1.92 a	191.77 ± 3.86 d	195.77 ± 3.86 d	204.76 ± 8.89 c	201.16 ± 3.16 cd
shoots	NA	NA	NA	NA	49.59 ± 2.44 a	52.65 ± 1.51 a

(TPC; µg epicatechin equivalents per mg dry weight) in grapevine tissues after chitosan application before and after veraison. All data were obtained from three biological replicates; means ± standard deviations within a row followed by different letters (a–f) are statistically different (*p* < 0.05; ANOVA Turkey’s test). NA = not analyzed.

**Table 3:** The total anthocyanin content.

Variety/Tissue	Harvesting time/Treatment					
	During Veraison		After Veraison		At Complete Maturation	
	Control	Chitosan	Control	Chitosan	Control	Chitosan
‘Touriga Franca’						
berry seeds	0.00 ± 0.00 a	0.00 ± 0.00 a	0.00 ± 0.00 a	0.00 ± 0.00 a	0.00 ± 0.00 a	0.00 ± 0.00 a
berry skins	16.52 ± 0.44 e	22.35 ± 0.91 cd	20.86 ± 0.87 d	24.49 ± 0.34 ab	26.02 ± 1.02 a	23.28 ± 0.55 bc
cluster stems	NA	NA	NA	NA	8.26 ± 0.02 b	17.66 ± 0.01 a
leaves	0.39 ± 0.02 b	0.50 ± 0.01 a	0.32 ± 0.02 c	0.44 ± 0.01 ab	0.39 ± 0.01 b	0.53 ± 0.01 a
shoots	NA	NA	NA	NA	1.55 ± 0.11 a	1.12 ± 0.02 a
‘Tinto Cão‘						
berry seeds	0.00 ± 0.00 a	0.00 ± 0.00 a	0.00 ± 0.00 a	0.00 ± 0.00 a	0.00 ± 0.00 a	0.00 ± 0.00 a
berry skins	5.32 ± 0.19 f	8.30 ± 0.40 e	21.16 ± 1.32 d	23.39 ± 0.25 c	28.74 ± 0.97 b	35.16 ± 1.16 a
cluster stems	NA	NA	NA	NA	2.25 ± 0.01 b	2.68 ± 0.02 a
leaves	0.76 ± 0.06 b	0.93 ± 0.04 a	0.67 ± 0.01 c	0.77 ± 0.03 b	0.78 ± 0.03 b	0.77 ± 0.03 b
shoots	NA	NA	NA	NA	0.14 ± 0.02 a	0.15 ± 0.01 a

(TAC; µg malvidin-3-*O*-glucoside equivalents per mg dry weight) in grapevine tissues after chitosan application before and after veraison. All data were obtained from three biological replicates; means ± standard deviations within a row followed by different letters (a–f) are statistically different (*p* < 0.05; ANOVA Turkey’s test). NA = not analyzed.

**Table 4:** The total tannin content.

Variety/Tissue	Harvesting time/Treatment					
	During Veraison		After Veraison		At Complete Maturation	
	Control	Chitosan	Control	Chitosan	Control	Chitosan
‘Touriga Franca’						
berry seeds	33.19±4.57 e	141.11 ± 5.95 a	48.23 ± 5.28 d	124.93 ± 14.32 b	95.35 ± 5.32 c	125.98 ± 4.54 b
berry skins	74.24 ± 3.68 b	84.78 ± 4.78 a	59.72 ± 3.78 c	82.09 ± 6.41 a	47.41 ± 3.02 d	57.11 ± 0.76 c
cluster stems	NA	NA	NA	NA	31.63 ± 3.11 a	20.52 ± 2.28 b
leaves	41.75 ± 5.42 a	36.90 ± 1.11 b	35.97 ± 3.45 b	32.47 ± 2.33 c	17.06 ± 1.15 d	32.47 ± 3.37 c
shoots	NA	NA	NA	NA	17.26 ± 1.62 b	37.02 ± 0.66 a
‘Tinto Cão‘						
berry seeds	129.04 ± 5.34 b	140.95 ± 2.64 a	108.50 ± 1.86 c	131.63 ± 5.54 b	106.81 ± 1.23 c	113.64 ± 4.25 c
berry skins	43.69 ± 3.61 b	47.89 ± 2.14 a	26.47 ± 5.07 d	40.40 ± 0.60 b	32.03 ± 4.17 c	47.55 ± 9.57 a
cluster stems	NA	NA	NA	NA	102.66 ± 5.28 b	140.65 ± 8.37 a
leaves	64.78 ± 10.10 c	88.19 ± 1.92 a	62.67 ± 3.86 c	77.53 ± 3.86 b	89.43 ± 8.89 a	78.72 ± 3.16 b
shoots	NA	NA	NA	NA	30.75 ± 2.44 b	37.97 ± 1.51 a

(TTC; µg epicatechin equivalents per mg dry weight) in grapevine tissues after chitosan application before and after veraison. All data were obtained from three biological replicates; means ± standard deviations within a row followed by different letters (a–e) are statistically different (*p* < 0.05; ANOVA Turkey’s test). NA = not analyzed.
